# Case Report: A recurrent case of ALK-ALCL after autologous transplantation was successfully treated with BV + a modified CHEP chemotherapy containing mitoxantrone hydrochloride liposome with the addition of chidamide maintenance therapy

**DOI:** 10.3389/fonc.2023.1242552

**Published:** 2023-10-02

**Authors:** Zhen Shang, Qi Zhang, Wanying Liu, Jiaying Wu, Yicheng Zhang, Yi Xiao

**Affiliations:** ^1^ Department of Hematology, Tongji Hospital of Tongji Medical College, Huazhong University of Science and Technology, Wuhan, China; ^2^ Immunotherapy Research Center for Hematologic Diseases of Hubei Province, Tongji Hospital of Tongji Medical College, Huazhong University of Science and Technology, Wuhan, China

**Keywords:** bone involvement, ALK-negative anaplastic large cell lymphoma, mitoxantrone hydrochloride liposome, chidamide, brentuximab vedotin

## Abstract

**Background:**

ALK-negative anaplastic large cell lymphoma (ALK-ALCL) is a rare heterogeneous malignancy of T-cell origin.ALK- ALCL has a poor prognosis, with more patients experiencing relapses and refractory to treatment, and its treatment remains challenging. We report a case with bone involvement as the main clinical manifestation of recurrent, and the patient achieved significant partial remission after brentuximab vedotin(BV) combined with a modified CHEP chemotherapy containing mitoxantrone hydrochloride liposome (PLM60) with the addition of chidamide maintenance therapy and received regular follow-up, with a disease-free survival of 16 months to date. A literature review of the clinical presentation and treatment of ALCL was also conducted to identify strategies for its diagnosis and management.

**Conclusions:**

ALK-ALCL with bone involvement as the main manifestation of recurrent is relatively rare. Here, BV combined a modified CHEP chemotherapy containing mitoxantrone hydrochloride liposome was applied for the first time in a patient with relapsed ALK-ALCL, inducing remission and extending survival. However, further prospective studies with many patients are needed to determine the biological characteristics of this rare type of ALK-ALCL and relevant treatment strategies.

## Introduction

ALK-negative anaplastic large cell lymphoma (ALK-ALCL) is a type of CD30+ peripheral T-cell lymphoma (PTCL) that was defined by the World Health Organization in 2008; ALK-ALCL is characterized by ALK negativity, accounting for 2-3% of non-Hodgkin lymphoma (NHL) and 12% of T-cell NHL cases (1, 2). Patients with ALK-ALCL often present with advanced disease (stage III-IV) and have type B symptoms, high international prognostic index (IPI) scores, elevated serum lactate dehydrogenase (LDH) levels, and an aggressive clinical course (3). Approximately 50% of ALK-ALCL cases show lymph node involvement, while extranodal involvement is less common. Extranodal involvement is most common in the skin, soft tissues, liver, and lungs. Skin lesions usually present as papules, nodules, or tumors (4–6). Recurrence with prominent bone damage and skin ulcers is relatively rare.

ALK-ALCL has a poor prognosis, with more patients experiencing relapses and refractory to treatment, and its treatment remains challenging. The LYSA/SFGMTC study reported that the survival of 74 adult patients with relapsed/refractory (R/R) ALK-ALCL was 5.3 and 8.1 months with chemotherapy alone (7). Some researchers have modified conventional chemotherapy regimens to improve treatment outcomes (8). Mitoxantrone hydrochloride liposome (PLM60) is a new drug to be marketed in China in 2022. The 2022 CSCO guidelines recommend the use of PLM60 in R/R PTCL based on efficacy and safety data from a pivotal phase 2 study (9, 10). Hematopoietic stem cell transplantation (HSCT) is also recommended in patients with R/R ALK-ALCL (11, 12). Molecular targeted agents, including brentuximab vedotin (BV) and histone deacetylase inhibitors (romidepsin and chidamide), hold some promise for R/R ALK-ALCL. BV was approved for R/R ATCL in 2011 and is increasingly being used to induce remission in relapsed patients (13–15). Romidepsin is recommended in treatment for relapsed or refractory peripheral and cutaneous T-cell lymphoma (16, 17). Chidamide, a comparable drug to romidepsin, also improves CR rates in R/R PTCL, including ALK-ALCL (18–20). Currently, some other molecular targeted drugs, such as Cergulatinib, Duvelisib, Tolinapant, and Golidocitinib, are in phaseI/II clinical research and have not shown significant therapeutic efficacy of a single drug in R/R PTCL. However, it is expected that the combination therapy will synergistically enhance the efficacy of R/R PTCL. To date, the treatment of R/R ALK-ALCL remains a challenge due to the lack of large-scale multicenter randomized clinical trials.

The purpose of this study was to report a case with prominent bone damage as a rare clinical manifestation of R/R ALK-ALCL; the patient was treated with BV combined with modified CHEP chemotherapy (containing PLM60) and chidamide for the maintenance therapy, which improved progression-free survival (PFS). The available scientific literature was also reviewed and summarized to improve clinician understanding of ALK-ALCL and provide a new treatment for this type of ALK-ALCL.

## Case

**Figure 1:**
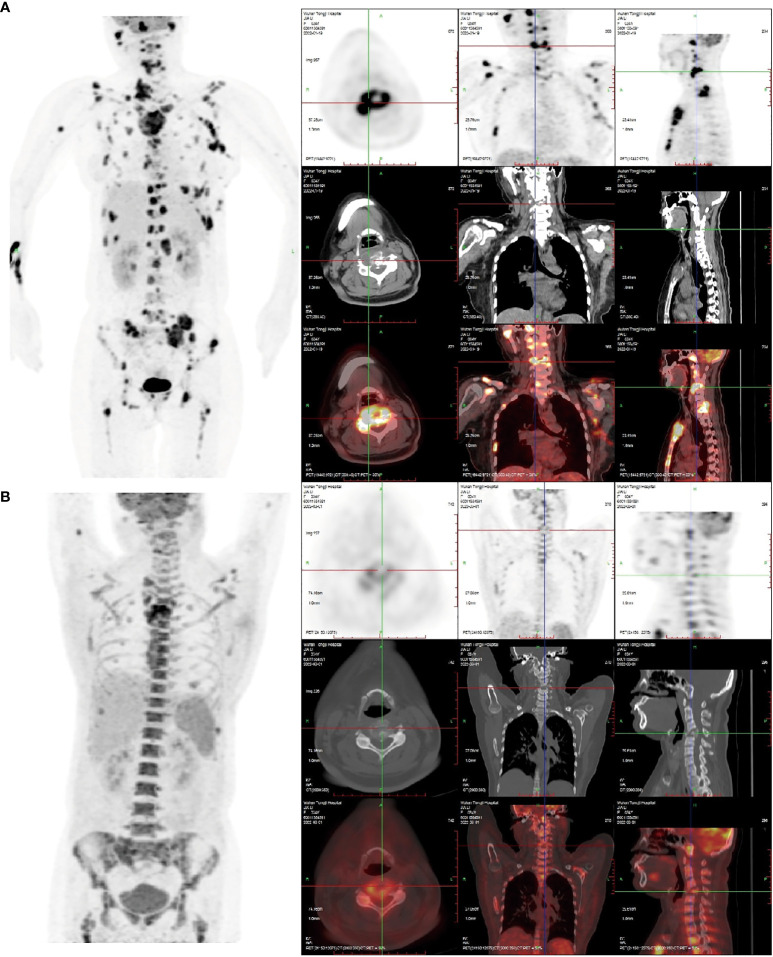
**(A)** The 18F-FDG PET/CT image on January 20, 2022: Right frontal bone, jaw bone, occipital slope, right humerus, sternum, bilateral clavicles, bilateral shoulder blades, bilateralmultiple ribs, extensive cervical/thoracolumbar vertebrae appendages, pelvic bones, and bilateralfemurs were involved. Some lesions were associated with soft tissue masses, with significantlyincreased radiotracer uptake. The size of the soft tissue masses in the sternum region wasapproximately 5.5 cm×2.9 cm×3.8 cm, and the SUVmax was 16.8. Increased and enlargedlymph nodes were seen in the right parotid gland region, right supraclavicular region, and leftarmpit, with increased radiation uptake. The largest lymph node was in the left armpit(approximately 1.6 cm × 1.4 cm in size, SUVmax 15.6.). **(B)** The 18F-FDG PET/CT image of thepatient after receiving two courses of chemotherapy on March 19, 2022. Compared with the **(A)** image, in the **(B)** image shown, there was a significant reduction or disappearance of radioactivityuptake in multiple bones and multiple lymph nodes, including the virtual disappearance of thesoft tissue mass in the sternal region, and a reduction of radioactivity uptake in the adjacentsternum compared with the previous one (SUVmax: 10.6). The left axillary lymph node lesiondisappeared without significant radioactivity uptake. Furthermore, bone fractures exist, and thebone marrow metabolic activity is increased (likely due to the effect of colony-stimulatingfactors).

**Figure 2:**
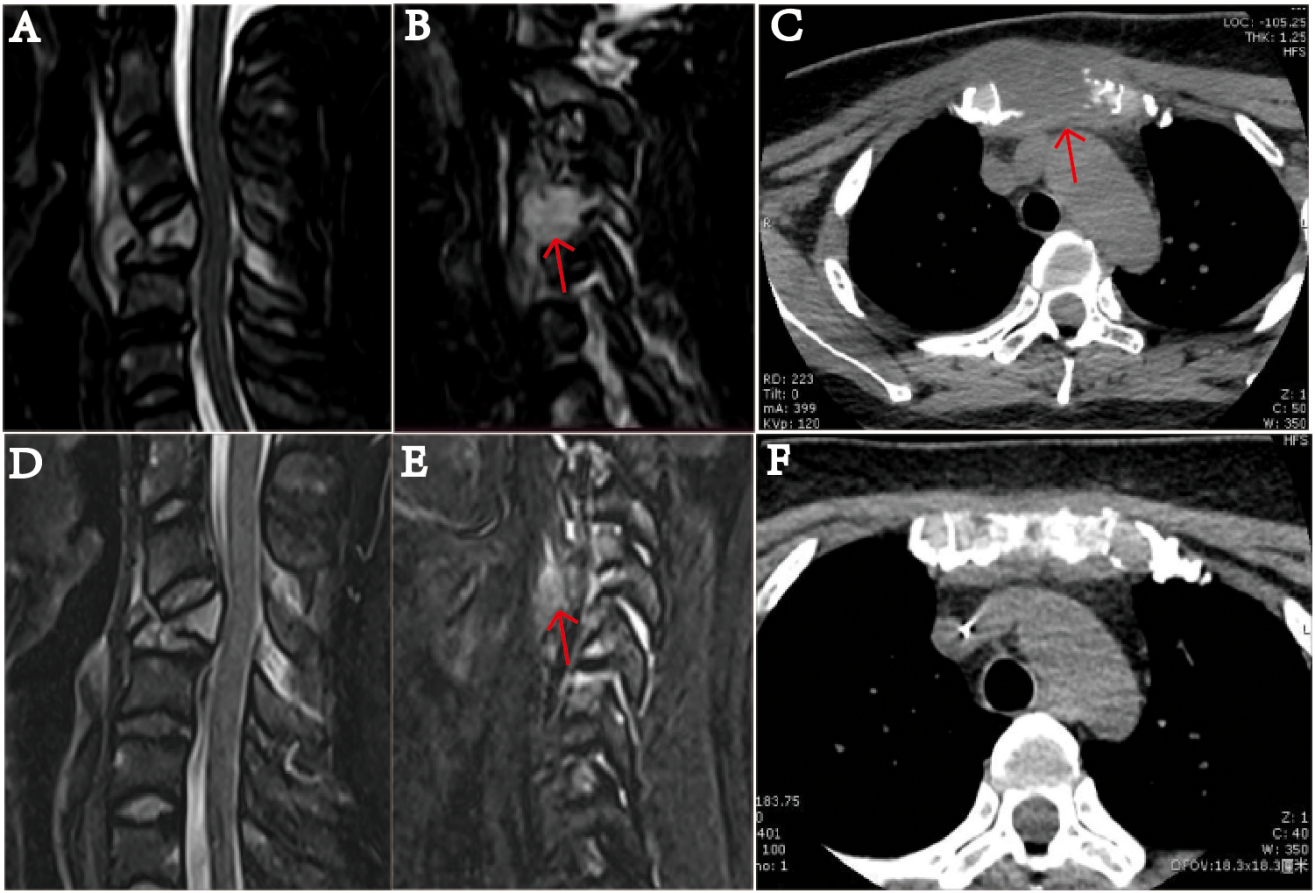
MRI examination of the cervical vertebra of the patient on January 20, 2022. **(A)** shows slope, C4, T1 vertebrae, and ancillary multiple bony lesions with pathologic fracture of the C4 vertebrae. **(B)** shows a 1.49 mm ×1.54 mm mass (indicated by the arrow) near the C4 cone, and the C4 vertebral body is compressed and flattened and shows pathological fracture. **(C)** The sternum is fractured, and there is a 37.41 mm × 31.14 mm soft tissue mass (indicated by the arrow). On March 19, after receiving two courses of chemotherapy, the patient showed destruction of multiple bones and a cervical spine fracture on MRI **(D–F)**. In part **(E)**, the C4 paravertebral mass is significantly reduced (indicated by the arrow). In part **(F)**, the soft tissue mass causing the sternum bone fracture is essentially undetectable.

**Figure 3:**
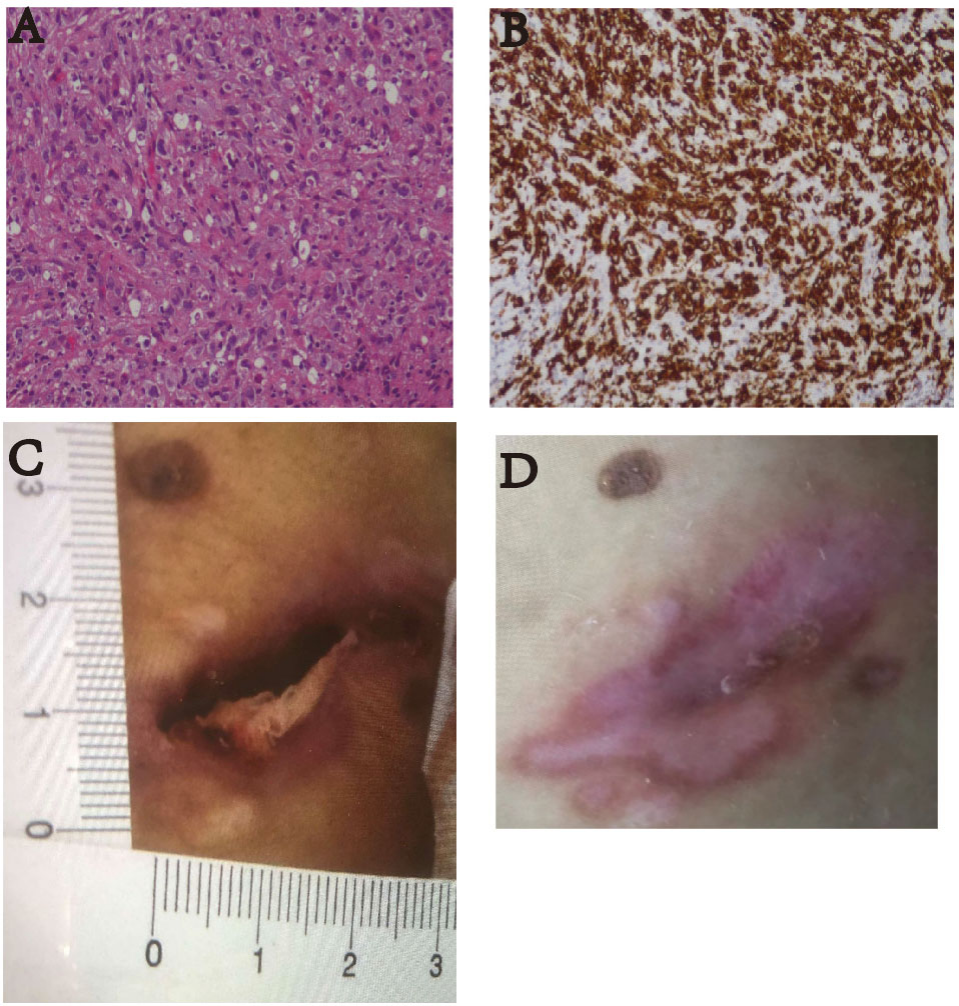
**(A)** Pathological HE image of the patient’s lymph node puncture (100×). The results suggested anaplastic large cell lymphoma (ALK-negative). **(B)** The immunohistochemistry image shows CD30 staining (100×). Diffuse enhancement of CD30 was seen. **(C)** On January 17, 2022, the patient had skin ulcers on the back of his waist (approximately 1.0 cm × 3.0 cm). **(D)** After receiving two courses of treatment, the skin ulcers healed on March 20, 2022.

**Figure 4:**
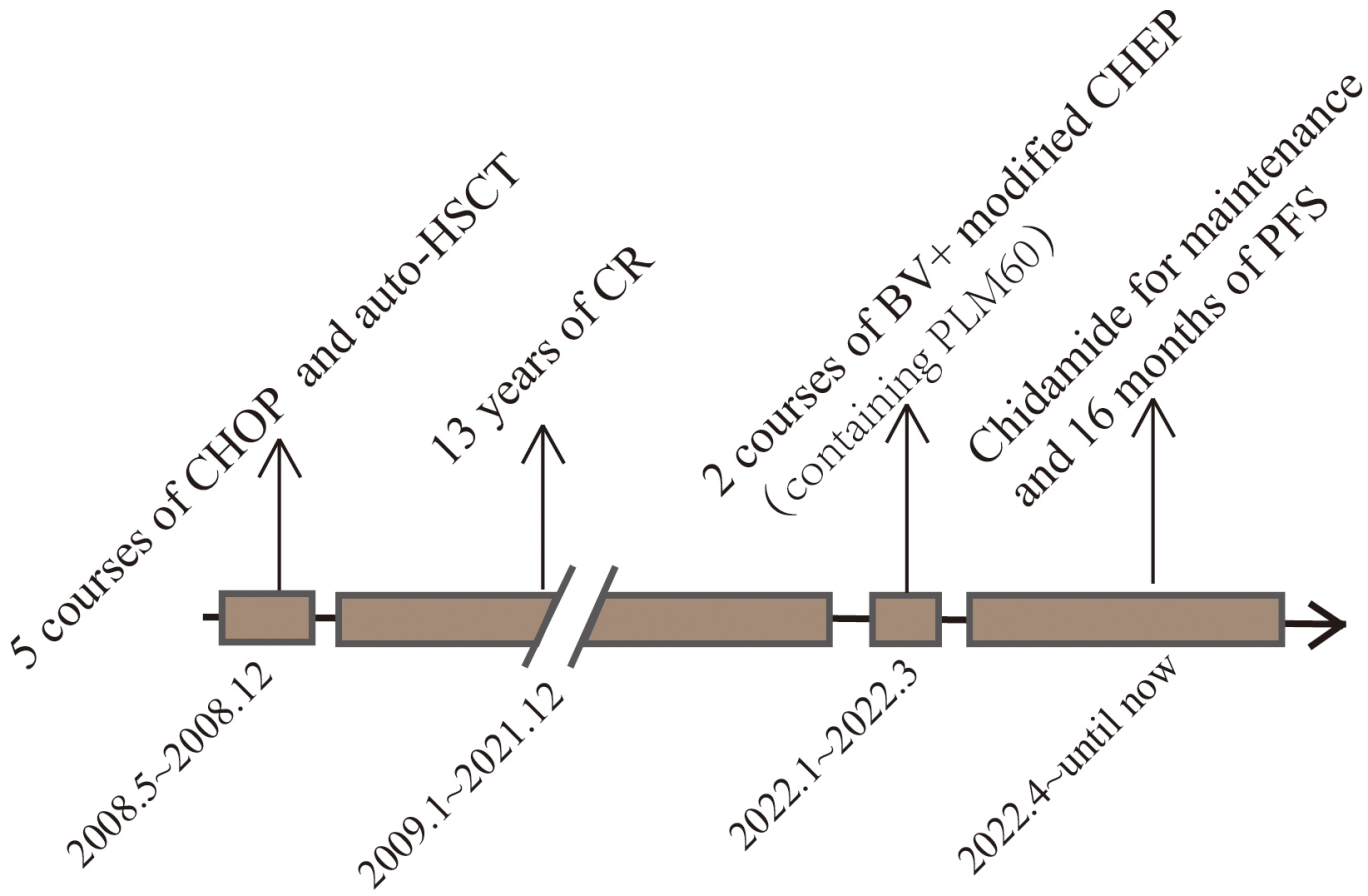
This image represents the patient’s treatment and status according to the timeline from the onset of the disease to the present.

A female patient of Chinese Han nationality was diagnosed with ALK-ALCL when she was 21 years old, due to “recurrent skin breakdown and enlarged right axillary lymph nodes for 1 week”. The patient received 5 courses of CHOP chemotherapy, and an autologous hematopoietic stem cell transplant after the first remission and achieved a disease-free survival of 13 years. She was readmitted to the hospital on January 15, 2022, when she was 34 years old with neck pain and mild numbness in her left limb. The patient’s history was free of relevant diseases. The patient’s family history and genetic history are free of genetically related diseases and free of hematologic disorders such as lymphoma. Physical examination revealed pressure-based pain in the neck and shoulders, head immobility, mild numbness in the left limbs, slightly decreased muscle strength, multiple positive lymph nodes (1.5cm×1.5cm) in the axilla and neck area, and a skin ulcer of approximately 1.0cm×3.0cm in the posterior lumbar region. Laboratory tests revealed a significant increase in blood calcium. PET/CT on January 20, 2022, showed destruction of multiple bones (including right frontal bone, jaw bone, occipital slope, right humerus, sternum, bilateral clavicles, bilateral shoulder blades, bilateral multiple ribs, extensive cervical/thoracolumbar vertebrae appendages, pelvic bones, and bilateral femurs) and lymph node enlargement, and lymphoma recurrence was suspected (see **Figure 1** for details). MRI of the cervical spine on January 20, 2022, revealed the following: slope, C4, T1 vertebrae, and ancillary multiple bony lesions with pathologic fracture of the C4 vertebrae, C4 paravertebral and C7/T1 right para-annexal soft tissue mass (see **Figure 2** for details). The pathological diagnosis based on the evaluation of the lymph nodes (axillary) was ALCL (ALK-negative), CD30 (diffuse+) (**Figure 3**), GrB (+), CD2 (weak +), CD8 (+), CD25 (+), MUM1 (+), ALK (-), CD4 (-), CD43 (-), CD3 (-), CD5 (-), PAX5 (-), CD19 (-), EMA (weak+), P63 (-), LEF1 (-), and ki67 (+ in approximately 80% of cells). Bone marrow examination did not show lymphoma involvement. Second-generation sequencing of lymphoma tissue DNA identified the following aberrations: 1.DDX3X missense mutation 11 (NM_001356.4 (DDX3X): c.1042G>A; p.Glu348Lys; mutation abundance 40.3%); 2. CREBBP missense mutation 31 (NM_004380.2 (CREBBP): c.5286G>T; p.Lys1762Asn; mutation abundance 35.4%); 3. DTXI non-shift deletion 2 (NM_004416.2 (DTX1): c.696_698del; (p. Pro238del; mutation abundance 2.3%); and 4. ARIDIA non-shift deletion (NM_006015.4 (ARIDIA): c.114_116del; p.Ala45del; mutation abundance 2.0%). The results of FISH analysis of lymphoma tissue sections were as follows: No ALK, CMYC, or IRF4 gene break rearrangement, no P53 gene deletion, and a low percentage of tetraploid cells. The patient was initially diagnosed with recurrence of ALCL (ALK-negative, CD30+) stage IV, IPI score 3 (bone and skin involvement), and hypercalcemia. The patient was administered the following regimen twice: 100 mg BV + a modified CHEP regimen (cyclophosphamide 1200 mg d1, PLM60 20 mg d1, etoposide 100 mg d1-d4 and methylprednisolone 80 mg dl-d4) on January 26, 2022. After the two chemotherapy cycles, a review was performed on March 19, 2022. Physical examination reveals shrinking lymph nodes and healing skin ulcers, as shown in **Figure 3**. PET/CT showed almost complete disappearance of the soft tissue mass and positive lymph nodes in the sternal region, as shown in **Figure 1**. The MRI examination of the cervical spine revealed the following: the destruction of multiple bones in the cervical spine and fracture, the disappearance of the paravertebral mass, and parasternal mass, as shown in **Figure 2**. On March 25, the patient underwent cervical fusion with anterior cervical decompression surgery. The results of the postoperative bone histopathology analysis were as follows: scattered brittle bone and cartilage tissue with localized fibrous tissue hyperplasia between the bones and no lymphoma component. The patient refused follow-up chemotherapy due to financial reasons. The patient received oral chidamide 5 mg qd from January 26, 2022, to the present, both during chemotherapy and maintenance therapy. To date, the patient has survived disease-free for 16 months (**Figure 4**).

## Discussion

The WHO 2016 classification identifies four ALCL entities: ALK+ALCL, ALK- ALCL, primary cutaneous ALCL, and breast implant-associated ALCL (BIA ALCL) (2). ALK-ALCL can occur in all age groups, with a peak incidence at 40~65 years, a slight male predilection, a median age of 56 years, and a median age of 31.5 years (3–5).

In 1991, Chan JK reported two cases of ALCL, one in a 22-year-old male with paralysis due to thoracic vertebral involvement who disease-free for 42 months after five courses of CHOP treatment and local radiotherapy and one affecting a 22-year-old male with mild bone destruction in the right acetabulum. The second patient developed right hip pain and received six courses of bleomycin+CHOP chemotherapy and local radiotherapy and survived disease-free for 34 months (21). In 1995, Tian C reported the case of a 20-year-old ALCL patient with thoracic spine involvement resulting in back pain and incomplete paralysis who died after 14 months (22). The ALK status of the above three patients could not be identified. In 2015, the case of a 40-year-old female with left hip involvement as a clinical manifestation of ALK+ALCL was reported. She was treated with Hyper-CVAD/MA for six cycles to achieve CR and survived disease-free for 24 months (23). To date, there are no clinical reports of adult ALK-ALCL with predominant bone involvement at relapse.

There are many studies on factors related to the prognosis of ALK-ALCL. The factors affecting prognosis include age, serum LDH level, β2 microglobulin level, time to relapse or progression after first treatment, extranodal involvement, and histological type. Naoko Tsuyama et al. summarized previous studies and concluded that there was no significant difference in prognosis between ALK- ALCL and ALK+ ALCL before the age of 40 years. After the age of 40 years, the prognosis of ALK- ALCL was found to be worse than that of ALK+ ALCL (3, 24). In a study by Xiu-Wen Deng et al. analyzing 48 patients with ALK- ALCL and 119 patients with PTCL-NOS, elevated LDH, ≥2 extranodal sites, and advanced disease were unfavorably associated with OS and PFS in ALK- ALCL (25). In the present case, regarding the clinical data, the patient was younger than 40 years old at the time of relapse and had normal LDH, which are indicators of a better prognosis.However, she also had extranodal sites ≥2 and advanced disease, which are associated with poor prognosis.

Standard treatment is intensified chemotherapy with or without ASCT consolidation, unfortunately, there are no effective treatments in Alk-ALCL.Until now, CHOP, CHOP-like(cyclophosphamide, adriamycin, vincristine, prednisone + etoposide or isocyclophosphamide) or hyperCVAD/MA regimens, are widely used clinically as the initial treatment regimen for ALK-ALCL (26). Whether to add ASCT treatment for the first remission (CR1) used to be controversial. In 117 patients diagnosed with PTCL(including 31 ALCL-ALK), a significant benefit of first-line autologous SCT(ASCT) over non-transplanted induction therapy was not identified (27). In 119 patients (83 with ASCT and 36 without ASCT) with nodal PTCL in CR1, the group with ASCT was associated with superior survival for patients with advanced-stage disease, intermediate-to-high IPI scores, or AITL subtype (28). However, recently, a large multicenter international retrospective study from Spain and Italy confirmed that ASCT could be a consolidation of first-line chemotherapy in patients with PTCL. After a median follow-up of 65.5 months in 174 patients with PTCL (101 with ASCT and 73 without ASCT), the PFS was slightly better in the transplanted group than in the non-transplanted group (5-year PFS rate 63% vs. 49%); in addition, the OS rate was also better in the transplanted group (5-year OS rate 74% vs. 62%) (29). The patient we reported was given ASCT after CR1 and achieved a disease-free survival of 13 years.

CHOP regimens or modified CHOP regimens in R/R PTCL have also been discussed (30, 31).PLM60 is a relatively new drug available in China. The new drug has PEG added to its surface, which allows it to escape immune surveillance and prolongs its circulation time, achieving a half-life of up to approximately 90 hours and promoting drug accumulation near tumor tissues and passive targeting of tumors (32–34). A single-arm, open-label, multicenter phase II study of PLM60 in R/R PTCL (including 14 cases of ALK-ALCL) and NK/T-cell lymphoma with 108 patients employed the objective remission rate (ORR) as the primary endpoint. The ORR was 41.7%, and the CR rate was 23.1%. The longest disease-free duration was 19.45 months. The median PFS was 8.5 months, and the median OS was 22.8 months (10).

An increasing number of new therapeutic approaches are being explored for patients with R/R PTCL. The four FDA-approved novel monotherapy agents are pralatrexate, BV, romidepsin, and belinostat (35). Pralatrexate, the first FDA-approved drug for R/R PTCL, is a novel folate antagonist, and pralatrexate achieved an ORR of 35% in 17 patients with ALCL in a prospective study of patients with R/R PTCL (36). BV is widely used for initially diagnosed and R/R PTCL (37, 38). The double-blind randomized phase 3 ECHELON-2 study demonstrated improved PFS and OS with frontline BV+CHP (37). There have been additional studies about BV on R/R T-cell lymphoma, with objective remission rates (ORRs) of 41% ~ 86% and median PFS values of 2.6~20.0 months (12, 39, 40). In patients with CD30+ R/R ALCL, BV is a reasonable option, but the prognosis after relapse is particularly poor, with a median OS < 2 months (41). CD30 deficiency in R/R T-lymphoblastic lymphoma may account for the poor outcome of BV (42). Histone deacetylase inhibitors such as romidepsin and belinostat have performed well in the treatment of R/R PTCL. A multicenter phase II study in 130 patients with R/R PTCL (ALK- ALCL, n = 21 (16%)) found that the addition of romidepsin significantly improved the CR rate (16). Chidamide, another histone deacetylase inhibitor used in this case, has also performed well in several trials (18, 19, 43). In a study of 383 patients with R/R PTCL in mainland China, for patients receiving chidamide monotherapy (n=256), the ORR and disease control rate (DCR) were 39.06 and 64.45%, respectively. For patients receiving chidamide in combination with chemotherapy (n =127), the ORR and DCR were 51.18% and 74.02%, respectively (18).

Therapeutically, after relapse in this case, BV combined with a modified CHEP chemotherapy containing PLM60 with the addition of chidamide maintenance therapy was effective. Significant partial remission was achieved with two courses of chemotherapy. In the later stage, although the patient did not undergo further treatment, he received oral chidamide maintenance therapy, and to date, the PFS is 16 months. Therefore, the new combination strategy could be tried in the R/R ALK- ALCL. However, the long latency of this case may not have relevance to other typical cases which relapse much earlier and are likely less therapy responses. Further prospective studies with many patients are needed to determine the biological characteristics of this rare type of ALK-ALCL and relevant treatment strategies.

## Patient perspective

13 years ago, I was treated by a doctor for lymphoma. After systematic treatment, I had a happy life for 13 years, during which I had my husband and my daughter. Last year, I had a recurrence of lymphoma which resulted in a fracture of my cervical vertebrae, skin ulcers and enlarged lymph nodes. After two courses of a new regimen of chemotherapy, orthopedic surgery and maintenance therapy with Cedarbenzamide, I am back to my normal life and work and am able to be there for my daughter’s upbringing.

## Data availability statement

The original contributions presented in the study are included in the article/supplementary material. Further inquiries can be directed to the corresponding authors.

## Ethics statement

The studies involving humans were approved by the Medical Ethics Committee of the Department of Hematology, Tongji Hospital, Tongji Medical College, Huazhong University of Science and Technology. The studies were conducted in accordance with the local legislation and institutional requirements. The participants provided their written informed consent to participate in this study. Written informed consent was obtained from the individual(s) for the publication of any potentially identifiable images or data included in this article.

## Author contributions

All authors designed the study, interpreted the findings and revised the manuscript. ZS, QZ, and JW carried out data management and statistical analysis and drafted the manuscript. WL helped with English language editing and data management. YZ and YX performed project administration. All authors contributed to the article and approved the submitted version.
